# A simple refined DNA minimizer operator enables 2-fold faster computation

**DOI:** 10.1093/bioinformatics/btae045

**Published:** 2024-01-25

**Authors:** Chenxu Pan, Knut Reinert

**Affiliations:** Department of Mathematics and Computer Science, Freie Universität Berlin, Takustraße 9, Berlin, 14195, Germany; Department of Mathematics and Computer Science, Freie Universität Berlin, Takustraße 9, Berlin, 14195, Germany; Max Planck Institute for Molecular Genetics, Ihnestraße 63-73, Berlin, 14195, Germany

## Abstract

**Motivation:**

The minimizer concept is a data structure for sequence sketching. The standard canonical minimizer selects a subset of *k*-mers from the given DNA sequence by comparing the forward and reverse *k*-mers in a window simultaneously according to a predefined selection scheme. It is widely employed by sequence analysis such as read mapping and assembly. *k*-mer density, *k*-mer repetitiveness (e.g. *k*-mer bias), and computational efficiency are three critical measurements for minimizer selection schemes. However, there exist trade-offs between kinds of minimizer variants. Generic, effective, and efficient are always the requirements for high-performance minimizer algorithms.

**Results:**

We propose a simple minimizer operator as a refinement of the standard canonical minimizer. It takes only a few operations to compute. However, it can improve the *k*-mer repetitiveness, especially for the lexicographic order. It applies to other selection schemes of total orders (e.g. random orders). Moreover, it is computationally efficient and the density is close to that of the standard minimizer. The refined minimizer may benefit high-performance applications like binning and read mapping.

**Availability and implementation:**

The source code of the benchmark in this work is available at the github repository https://github.com/xp3i4/mini_benchmark

## 1 Introduction

The minimizer concept is a data structure for sequence sketching. It is firstly introduced to the sequence analysis by Roberts *et al.* (2004) to reduce the storage requirements of biological sequence data. Then it was applied by many other applications in the field, such as sequence binning (Deorowicz *et al.* 2015), sequence compaction (Chikhi *et al.* 2016), sequence classification (Wood and Salzberg 2014), and read mapping (Li 2016, Jain *et al.* 2020, Büchler *et al.* 2023).

Given the sequence, the minimizer is the minimum *k*-mer of a predefined ordering scheme in a window of *w* consecutive *k*-mers. The minimizer performance relates to several key measurements. Schleimer *et al.*’s (2003) study defined the density of a *k*-mer selection scheme as the fraction of selected *k*-mers. Formally, denote < the ordering scheme and *X* the selected *k*-mers in the sequence *S*, whose size |S|≫w+k. The density of the selection scheme is given by

(1) $$\rho (X)=\frac{\left|X\right|}{\left|S\right|}$$

where |*X*|, |*S*| are the size of *X* and *S*. Since it was first introduced to measure the storage requirements, the selection schemes are supposed to select a set of *k*-mers that is as sparse as possible such that the storage requirements can be largely reduced. Novel selection schemes, such as Orenstein *et al.* (2016), Marçais *et al.* (2017), Jain *et al.* (2020), and (Zheng *et al.* 2021), are proposed to improve the minimizer density.

The *k*-mer repetitiveness is another minimizer measurement. It is measured by the *k*-mer frequency in practice. Formally, the frequency of a *k*-mer X=x in *S* is defined as its average occurrences in the sequence,

(2) $$v(X=x)=\frac{n(x)}{\left|S\right|}$$

where n(x) is the occurrence of *k*-mer X=x. Let *V* denote the random variable over possible *k*-mer frequencies. It relates to the performance of applications such as

1. Read mapping: Consider the anchoring (seeding) problem, where we need to find all matched pairs of minimizers in the reference and the read.
2. Binning: Similar to the read mapping, we need to find matched minimizers and cluster them into bins.

For the two problems, we prefer selection schemes that can generate minimizers of lower repetitiveness (Deorowicz *et al.* 2015), because highly repetitive minimizers would significantly decrease the matching accuracy and computational efficiency.

Like many other fundamental data structures, computational efficiency is the third performance measurement. Although the time complexity of computing minimizers is commonly linear, optimizations of density or *k*-mer repetitiveness may significantly increase the runtime. For high-performance applications, such as population-scale read mapping, drops in computational efficiency may be non-negligible.

In general, there exist performance trade-offs between minimizer variants. For instance, the random ordering scheme (Chikhi *et al.* 2014) generates more uniformly and sparsely distributed minimizers than the lexicographic ordering scheme at the expense of increased runtime. In contrast, lexicographical minimizers are less affected by nearby mutations or sequencing errors than random minimizers, sometimes called “conservation” (Edgar 2021). Thus, they are beneficial to some matching applications. But the trade-off is the less random sampling.

Here, we propose an operator as a refinement of the standard (canonical) minimizer. It has the following features.

1. It improves *k*-mer repetitiveness of the standard minimizer. It is less biased to small *k*-mers and distributes more uniformly.
2. It applies to any selection schemes of total orders (Davey *et al.* 2002) (e.g. lexicographic or random order).
3. Its density converges toward that of the standard minimizers.
4. It is commonly faster than the standard minimizer to compute and can reach two times at most.

It is worth noting that the operator does not apply to non-canonical minimizers of single-strand sequences, such as RNA minimizers. However, canonical minimizers are essential to most sequence analysis applications, such as read mapping and genome assembly.

In the following sections, we will first define the refined minimizer. Next, we will prove three properties that are essential to the refined minimizer performance. In the results, we will compare the algorithm complexity of computing the standard and refined minimizers. Then, we will evaluate the statistics (e.g. repetitiveness, density) of standard and refined minimizers in real sequences. Finally, we will analyze the statistics and discuss the potential limitations and improvements.

## 2 Materials and methods

### 2.1 Definitions

*Operations:* For high-performance applications, a preferable minimizer function should be simple and effective. Specifically,

1. Simple: It uses a few operations to compute, such as operations in {+,−,≪,≫,&,|,⊕,CMP}, namely Add, Subtract, Bitwise Shift left/right, And, Or, Exclusive Or (XOR) and Branch Conditions.
2. Effective: It generates less biased *k*-mers with reasonable density. And it applies to all selection schemes.

Table 1 is a reference comparison of CPU cycles for operations we used to compute minimizers. It is dominated by branch conditions $o_{3}$, which takes about 10 cycles on average.

**Table 1.** CPU cycles for operations used to compute minimizers. Operations such as traversing an array will probably trigger $L_{1}$ cache read.

No.	Operations	CPU cycles
$o_{1}$	Add, Subtract, OR, AND, XOR, Shift	<1
$o_{2}$	Level 1 ($L_{1}$) cache read	3–4
$o_{3}$	Right “if” branch	1–2
	Wrong “if” branch (branch misprediction)	10–20

*Standard minimizer*: A minimizer scheme denoted by (w,k,<) selects the minimum *k*-mer in *w* consecutive *k*-mers $\in \Sigma^{k}$, where Σ is the character set and order < is commonly induced by a hash function *h*, which is an injection from $\Sigma^{k}$ to a totally ordered set. Namely, if *x*, *y* are two *k*-mers, then x<y if and only if h(x)<h(y). Denote *s* the subsequence (or window) whose length |s|=w + k − 1. Denote $s^{\prime }$ the reverse complement of *s*. The standard minimizer $h_{s}$ is given by

$$h_{s}(s)=\min_{0\;⩽\;i<w}^{<}\{s_{i,i+k},s_{i,i+k}^{\prime }\}$$

*Refined minimizer:* The core idea of the refined minimizer is to define an appropriate decision function that makes the ordering scheme only compute minimizers in the sequence of one strand such that the smallest *k*-mers are less likely to be selected, repetitively. Provided |s|≡1(mod2), we define an operator as

(3) $$\delta (s)=\begin{array}{c} p_{T}+p_{G}-p_{C}-p_{A} \end{array}$$

where $p_{A},p_{C},p_{G},p_{T}$ are the occurrences of characters *A*, *C*, *G*, *T* in *s*. |s|≡1 (mod2) is to guarantee δ(s)≠0, which will be later discussed in the properties. The refined minimizer *h* is then defined as

(4) $$h_{r}(s)=\{\begin{array}{cc} h_{r}^{-}(s)=\overset{<}{\underset{0⩽i<w}{\min}}\{s_{i,i+k}^{\prime }\} & \text{if}\;\delta (s)<0\\ h_{r}^{+}(s)=\overset{<}{\underset{0⩽i<w}{\min}}\{s_{i,i+k}\} & \text{if}\;\delta (s)>0 \end{array}$$

Table 2 is an example comparing refined and standard minimizers. The lexicographic order of a given *k*-mer can be computed by $\sum_{i=0}^{k-1}4^{i}a_{i}$, where $a_{i}$ is the order of the *i*th (right toward left) character of the *k*-mer and $a_{i}$ equals 0, 1, 2, 3 for *A*, *C*, *G*, *T*.

**Table 2.** Comparison of standard (Std) and refined (Rfd) minimizers in a DNA sequence *s* and reverse complement $s^{\prime }$, where |s|=11, k=5.

*n*	*s*′	*s*	δ(s′)	δ(s)	*K*		h(K)		$Q_{2}(h)$		max h − min h	
					Std	Rfd	Std	Rfd	Std	Rfd	Std	Rfd
1	AGCTTACTTTG	CAAAGTAAGCT	3	−3	AAAGT	ACTTT	11	127	11	**127**	**0**	**0**
2	GCTTACTTTGG	CCAAAGTAAGC	5	−5	AAAGT	ACTTT	11	127	11	**127**	**0**	**0**
3	CTTACTTTGGT	ACCAAAGTAAG	5	−5	AAAGT	ACTTT	11	127	11	**127**	**0**	**0**
4	TTACTTTGGTG	CACCAAAGTAA	7	−7	AAAGT	ACTTT	11	127	11	**127**	**0**	**0**
5	TACTTTGGTGT	ACACCAAAGTA	7	−7	AAAGT	ACTTT	11	127	11	**127**	**0**	**0**
6	ACTTTGGTGTT	AACACCAAAGT	7	−7	AAAGT	ACTTT	11	127	11	**127**	**0**	**0**
7	CTTTGGTGTTT	AAACACCAAAG	9	−9	AAACA	CTTTG	4	510	11	**127**	7	**383**
8	TTTGGTGTTTG	CAAACACCAAA	11	−11	AAACA	GGTGT	4	699	11	**127**	7	**572**
9	TTGGTGTTTGG	CCAAACACCAA	11	−11	AAACA	GGTGT	4	699	11	**127**	7	**572**
10	TGGTGTTTGGT	ACCAAACACCA	11	−11	AAACA	GGTGT	4	699	11	**127**	7	**572**
11	GGTGTTTGGTA	TACCAAACACC	9	−9	AAACA	GGTGT	4	699	11	**127**	7	**572**
12	GTGTTTGGTAA	TTACCAAACAC	7	−7	AAACA	GGTAA	4	688	11	**510**	7	**572**
13	TGTTTGGTAAA	TTTACCAAACA	5	−5	AAACA	GGTAA	4	688	4	**510**	7	**572**
14	GTTTGGTAAAT	ATTTACCAAAC	5	−5	ACCAA	GGTAA	80	688	11	**688**	76	**572**
15	TTTGGTAAATG	CATTTACCAAA	5	−5	AAATG	AAATG	14	14	11	**510**	76	**685**

*K* is the minimizer. h(K) is the lexicographic order of the minimizer. $Q_{2}(h)$ is the median of h(K). Values with bold text imply that *h* is less biased to small ones.

### 2.2 Properties

Here, we discuss three refined minimizer properties that are essential to the applications. They hold for all ordering schemes (w,k,<) defined above. The first one guarantees the strand symmetry, such that the computation of the refined minimizer is independent of the strand. The second one guarantees that the refined minimizer is always not smaller than the standard one. The third one guarantees that the refined minimizers have a reasonable density that is close to that of the standard one.

1. Provided |s|≡1 (mod2), then $h_{r}(s^{\prime })=h_{r}(s)$.
  Proof: Clearly, $p_{A}+p_{C}+p_{G}+p_{T}=|s|$.
  $$\begin{array}{c} \delta_{s^{\prime }}=p_{T}^{\prime }+p_{G}^{\prime }-p_{C}^{\prime }-p_{A}^{\prime }\\ =p_{A}+p_{C}-p_{T}-p_{G}\\ =-\delta_{s}=|s|-2(p_{G}+p_{T})\equiv 1\;\left(\mathrm{mod}2\right)\\ \neq 0 \end{array}$$
  where $p_{A}^{\prime }=p_{T}$, $p_{C}^{\prime }=p_{G}$, $p_{G}^{\prime }=p_{C}$, $p_{T}^{\prime }=p_{A}$ are the occurrences of *A*, *C*, *G*, *T* in $s^{\prime }$. Hence $h_{r}(s^{\prime })=h_{r}(s)$ according to the definition in expression 4.
2. For any total order < of $\Sigma^{k}$, $h_{s}(s)\;⩽\;h_{r}(s)$. Proof:
  $$\begin{array}{c} h_{s}(s)=\overset{<}{\underset{0⩽i<w}{\min}}\{s_{i,i+k},s_{i,i+k}^{\prime }\}\\ =\overset{<}{\min}\{\min_{0⩽i<w}^{<}\{s_{i,i+k}\},\min_{0⩽i<w}^{<}\{s_{i,i+k}\}\}\;⩽\;h_{r}(s) \end{array}$$
  It implies that $h_{r}(s)$ would be less biased to small *k*-mers than $h_{s}(s)$.
3. Denote $s_{n}=a_{0}a_{1},..,a_{|s|-1}$ and $s_{n+1}=a_{1}a_{2},..,a_{\left|s\right|}$ the *n*th and n+1th subsequences, where $a_{i}$ is the *i*th base. Denote $\delta_{n}=\delta (s_{n})$ the operator of $s_{n}$ defined in expression 3. Provided the sequence is random, then the following expression of probabilities holds
  (5) $$\begin{array}{c} \underset{|s|\to +\infty }{\lim}P\left(h_{r}\left(s_{n}\right)=h_{r}\left(s_{n+1}\right)\right)\\ =\underset{|s|\to +\infty }{\lim}P\left(h_{s}\left(s_{n}\right)=h_{s}\left(s_{n+1}\right)\right)\\ =1-\frac{2}{w+1} \end{array}$$
  Proof: For random sequences, Schleimer *et al.* (2003) have proved $P\left(h_{s}\left(s_{n}\right)=h_{s}\left(s_{n+1}\right)\right)=1-\frac{2}{w+1}$. Because there exist two cases that $h_{s}\left(s_{n}\right)\neq h_{s}\left(s_{n+1}\right)$, namely the minimizer of $s_{n}$ is its leftmost *k*-mer or the minimizer of $s_{n+1}$ is its rightmost one, otherwise $s_{n}$ and $s_{n+1}$ share the same minimizer. The probability of each case is $\frac{1}{w+1}$. Therefore, $P\left(h_{s}\left(s_{n}\right)=h_{s}\left(s_{n+1}\right)\right)=1-\frac{2}{w+1}$.

We then prove the limit of the refined minimizer in expression 5. Since $s_{n+1}$ can be iterated from $s_{n}$ by removing the first character of $s_{n}$, namely $a_{0}$, and append the last character of $s_{n+1}$, namely $a_{\left|s\right|}$, at the end, we have $\delta_{n+1}=\delta_{n}+d_{n}$, where

(6) $$d_{n}=\{\begin{array}{cc} -2 & \text{if}\;a_{0}\in \{G,T\}\;\text{and}\;a_{\left|s\right|}\in \{A,C\}\\ 0 & \text{if}\;a_{0},a_{\left|s\right|}\in \{G,T\}\;\text{or}\;a_{0},a_{\left|s\right|}\in \{A,C\}\\ 2 & \text{if}\;a_{0}\in \{A,C\}\;\text{and}\;a_{\left|s\right|}\in \{G,T\} \end{array}$$

It is worth noting that $\delta_{n}\delta_{n+1}\neq 0$, since δ≠0 has been proved in the first property. Then we have the following two cases:

- If $\delta_{n}\delta_{n+1}>0$: Then according to the definition in expression 4
  $$\begin{array}{c} P(h_{r}(s_{n})=h_{r}(s_{n+1})|\delta_{n}>0,\delta_{n+1}>0)\\ =P(h_{r}^{+}(s_{n})=h_{r}^{+}(s_{n+1}))=1-\frac{2}{w+1} \end{array}$$
  The probability above equals $1-\frac{2}{1+w}$ because there exist two cases that $P(h_{r}^{+}(s_{n})\neq h_{r}^{+}(s_{n+1})$ as well. Analogously,
  $$\begin{array}{c} P(h_{r}(s_{n})=h_{r}(s_{n+1})|\delta_{n}<0,\delta_{n+1}<0)\\ =P(h_{r}^{-}(s_{n})=h_{r}^{-}(s_{n+1}))=1-\frac{2}{w+1} \end{array}$$
  Therefore,
  $$\begin{array}{c} P(h_{r}(s_{n})=h_{r}(s_{n+1})|\delta_{n}\delta_{n+1}>0)\\ =P(h_{r}^{+}(s_{n})=h_{r}^{+}(s_{n+1}))\frac{P(\delta_{n}>0,\delta_{n+1}>0)}{P(\delta_{n}\delta_{n+1}>0)}\\ +P(h_{r}^{-}(s_{n})=h_{r}^{-}(s_{n+1}))\frac{P(\delta_{n}<0,\delta_{n+1}<0)}{P(\delta_{n}\delta_{n+1}>0)}\\ =1-\frac{2}{w+1} \end{array}$$
- If $\delta_{n}\delta_{n+1}<0$: $\delta_{n}\delta_{n+1}=\delta_{n}(\delta_{n}+d_{n})<0$ if and only if (iff) $d_{n}=\pm 2$ and $\delta_{n}\delta_{n+1}=-1$. According to expressions (3) and (6), we know that $\delta_{n}=-1$, $\delta_{n+1}=1$ iff $a_{0}\in \{A,C\}$, $\frac{|s|-1}{2}$ characters in $a_{1},a_{2},\ldots ,a_{|s|-1}$ are ∈{A,C} and $a_{\left|s\right|}\in \{G,T\}$. Therefore,
  $$\begin{array}{c} P(\delta_{n}=-1,\delta_{n+1}=1)=\frac{\left(\begin{array}{c} |s|-1\\ |s|-1 \end{array}\right)}{2}{\left(p(1-p)\right)}^{\frac{|s|+1}{2}} \end{array}$$
  where *p* is the probability of an random character ∈{A,C}. Analogously,
  $$P(\delta_{n}=1,\delta_{n+1}=-1)=\frac{\left(\begin{array}{c} |s|-1\\ |s|-1 \end{array}\right)}{2}{\left(p(1-p)\right)}^{\frac{|s|+1}{2}}$$
  The limits of the two probabilities above equal 0. Therefore, $\underset{|s|\to +\infty }{\lim}P(\delta_{n}\delta_{n+1}<0)=0$

Therefore, the limit in expression 5 is

$$\begin{array}{c} \underset{|s|\to +\infty }{\lim}P\left(h_{r}\left(s_{n}\right)=h_{r}\left(s_{n+1}\right)\right)\\ =\underset{|s|\to +\infty }{\lim}P(\delta_{n}\delta_{n+1}<0)P(h_{r}(s_{n})=h_{r}(s_{n+1})|\delta_{n}\delta_{n+1}<0)\\ +\underset{|s|\to +\infty }{\lim}P(\delta_{n}\delta_{n+1}>0)P(h_{r}(s_{n})=h_{r}(s_{n+1})|\delta_{n}\delta_{n+1}>0)\\ =1-\frac{2}{w+1} \end{array}$$

Based on the discussion above, we have the expected *k*-mer density of refined minimizers

(7) $$\rho_{r}=P(\delta_{n}\delta_{n+1}>0)\rho_{s}+P(\delta_{n}\delta_{n+1}<0)$$

where $\rho_{s}$ is the expected density of standard minimizers. Therefore, $\underset{|s|\to +\infty }{\lim}\rho_{r}=\rho_{s}$.

### 2.3 Heuristics

Expression (7) suggests that we can improve the *k*-mer density without significantly impacting the selected minimizers by simply skipping the n+1th window if $\delta_{n}\delta_{n+1}<0$. The core idea of the heuristic is to skip the “solo” windows, whose signs of δ are different from those of predecessor and successor windows. Solo windows are minority especially for large |*s*|, while they significantly increases $P(\delta_{n}\delta_{n+1}<0)$ in expression (7). The heuristic skips minimizers of solo windows while preserving minimizers of “non-solo” ones. For instance, if $\delta_{1},\delta_{2},\delta_{3},=-1,1,-1$, then skipping the solo window 2 will also drop its minimizer. However, if $\delta_{1},\delta_{2},\delta_{3},=-1,1,1$, then skipping window 2, which is non-solo, may not affect its minimizer, since window 3 may preserve it.

## 3 Results

### 3.1 Runtime

*Arbitrary windows:* We compared the CPU cycles of computing the refined and standard minimizer in algorithms 1 and 2. The loops in the pseudocodes apply to arbitrary windows and ordering schemes induced by the random hash function *R*, such as ntHash (Mohamadi *et al.* 2016), which directly computes random rolling hash values. CPU cycles for each step are listed in the comments of algorithms 1 and 2. Algorithm 1 takes $o_{r}=10o_{1}+2o_{2}+o_{3}+w(3o_{1}+o_{R}+o_{3})$ operations in sum and algorithm 2 takes $o_{s}=8o_{1}\;+\;o_{2}\;+\;2w(3o_{1}\;+\;o_{R}\;+\;o_{3})$ operations in sum, where $o_{1}$,…,$o_{3}$ are defined in Table 1, $o_{R}$ is CPU cycles for function *R*. Assuming $o_{1}=1$, $o_{2}=3$ and $o_{3}$ takes 10 cycles on average, then

$$\{\begin{array}{c} o_{s}=11+2w(13+o_{R})\\ o_{r}=26+w(13+o_{R}) \end{array}$$

The expected speedup of the refined minimizer is

(8) $$T_{r}=\frac{o_{s}}{o_{r}}=2-\frac{41}{26+w(13+o_{R})}$$

Hence, $T_{r}\in [0.949,2)$, where $T_{r}$ is minimized when w=1 and $o_{R}=0$ (lexicographic ordering). $T_{r}$ is maximized when w≫1 or $o_{R}\gg 0$. Therefore, the refined minimizer can be two times faster at most.

Applications may apply heuristics to further improve the minimizer performance. For instance, a more practical way to break ties (when the smallest *k*-mer appears multiple times) is to skip ties in adjacent windows. This creates optimal spread in poly-*X* regions (e.g. repetitive *AA*.). Such heuristics will introduce additional CPU cycles. However, heuristics for standard minimizers commonly apply to refined minimizers and can be integrated into function *R*. Hence the speedup upper bound can be preserved in such cases.

*Consecutive windows:* Applications may use buffers to reduce the times of computing *k*-mers when computing minimizers in consecutive windows. The refined minimizer preserves the speedup upper bound in such a case. They are discussed in Supplementary Notes. However, the speedup in practice can be washed out to some extent by additional buffer operations, such as reading, writing, traversing, etc. The exact trade-offs depend on *w*, *k*, ordering schemes, CPU architectures, etc. Optimizations of buffers can substantially improve the practical runtime in such cases.

**Figure:**
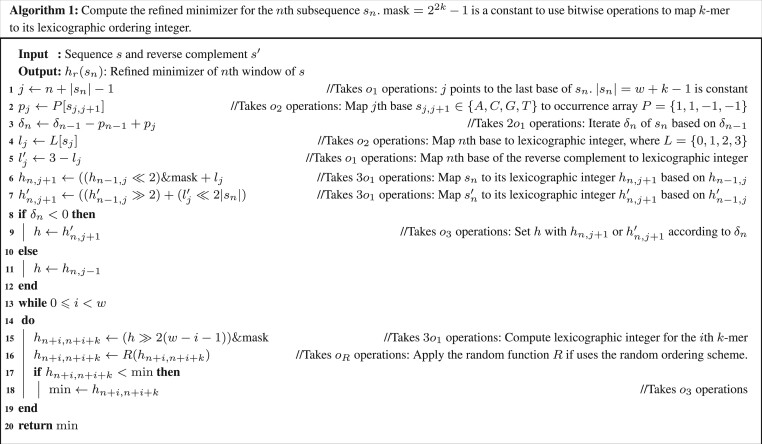


**Figure:**
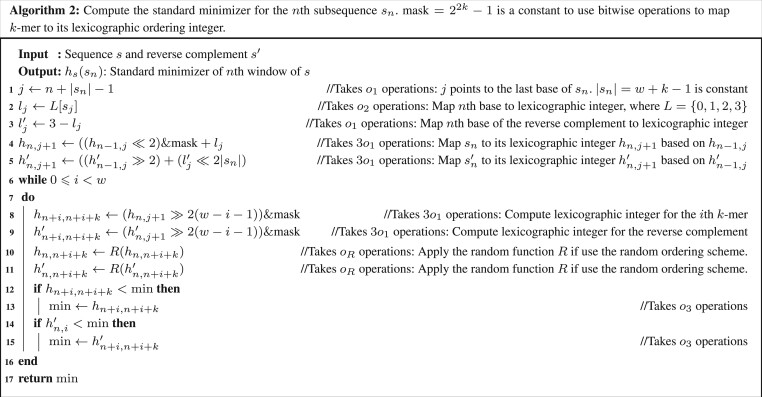


### 3.2 Distributions

As discussed above, we ideally prefer selection schemes that can generate *k*-mers of lower frequency for the read mapping and binning problem. Correspondingly, we prefer more uniformly distributed minimizers. We evaluated key statistics shown in Table 3 as a sketch of the distribution of selected minimizers *X*, which are computed in consecutive windows by streaming GRCH38 (chr 1–22, X, Y). Runtime (i.e. *T* in the table) is the corresponding time of computing minimizers in consecutive windows with buffers rather than the runtime of algorithms 1 and 2. Results for additional groups of |s| ⩽ 45 and k ⩽ 30 are presented in Supplementary Tables S1 and S2. It is worth noting that the tables only show statistics for even *k*s to simplify the results. The refined minimizer concept also applies to odd *k*s, and the corresponding results have no significant difference compared to those of even *k*s. Supplementary Table S3 shows statistics of minimizers of minimap2 (Li 2018). We evaluated 25–95% percentiles of minimizer frequency *V*, as shown in the table. For instance, $P_{0.25}=9.97$ per megabases for standard lexicographical minimizer with |s|=15,k=4 means 25% minimizer frequencies are lower than this value.

**Table 3.** Statistics of standard (Std) and refined (Rfd) minimizer sampled consecutively in GRCH38: $P_{0.25}$-$P_{0.95}$ are percentiles of minimizer frequency per megabases.

<	|s|,k	$P_{0.25}(V)$		$P_{0.5}(V)$		$P_{0.75}(V)$		$P_{0.95}(V)$		ρ(X)		$D_{KL}$		*E*-hits		*T* [s]	
		Std	Rfd	Std	Rfd	Std	Rfd	Std	Rfd	Std	Rfd	Std	Rfd	Std	Rfd	Std	Rfd
Lexico	15,4	9.97	**7.55**	201.92	**69.07**	2.64E3	**762.28**	1.01E4	**4.86E3**	**0.16**	0.21	2.28	**1.26**	2.53E7	**1.14E7**	27.60	**26.12**
	15,8	2.32	**0.06**	7.54	**0.40**	23.66	**2.58**	61.41	**25.48**	**0.26**	0.29	2.25	**1.59**	1.92E5	**1.23E5**	28.88	**26.62**
	15,12	0.01	**0.00**	0.02	**0.01**	0.09	**0.03**	0.28	**0.16**	**0.44**	0.46	2.43	**1.90**	1.29E4	**9.11E3**	31.03	**26.22**
	25,4	**0.28**	0.71	22.90	**4.34**	697.16	**170.95**	8.59E3	**3.88E3**	**0.09**	0.13	2.76	**1.73**	2.43E7	**1.19E7**	25.04	**22.73**
	25,8	0.04	**0.00**	1.04	**0.02**	7.27	**0.69**	44.77	**16.95**	**0.12**	0.16	3.01	**2.06**	2.26E5	**9.93E4**	25.03	**22.63**
	25,12	0.00	**0.00**	0.01	**0.00**	0.06	**0.01**	0.24	**0.12**	**0.16**	0.19	3.40	**2.67**	1.34E4	**8.32E3**	25.85	**23.02**
Random	15,4	7.82	**1.56**	163.57	**88.59**	1.74E3	**1.27E3**	7.92E3	**4.80E3**	**0.15**	0.19	2.14	**1.51**	2.26E7	**1.59E7**	48.57	**42.94**
	15,8	0.14	**0.04**	1.33	**0.48**	8.75	**3.70**	41.05	**25.83**	**0.22**	0.25	2.17	**1.67**	1.61E5	**1.26E5**	47.95	**43.21**
	15,12	0.00	**0.00**	0.01	**0.01**	0.06	**0.04**	0.23	**0.17**	**0.41**	0.42	2.31	**2.06**	1.07E4	**1.02E4**	52.04	**41.94**
	25,4	0.34	**0.07**	**6.32**	6.40	634.58	**472.81**	5.71E3	**3.48E3**	**0.09**	0.12	2.66	**2.01**	2.25E7	**1.71E7**	46.20	**38.61**
	25,8	0.01	**0.01**	0.24	**0.19**	3.62	**2.03**	31.74	**22.66**	**0.11**	0.14	2.83	**2.29**	1.61E5	**1.34E5**	45.89	**38.23**
	25,12	0.00	**0.00**	0.01	**0.00**	0.03	**0.02**	0.17	**0.12**	**0.14**	0.17	3.18	**2.74**	**8.97E3**	9.64E3	46.79	**41.85**

$D_{KL}(X||U)$ is the Kullback–Leibler (KL) divergence of the distribution of *X* and the uniform *k*-mer distribution *U*. Large values such as *E*-hits are expressed by scientific notation. *T* is the runtime. Better values are in bold text.

The column $D_{KL}(X||U)$ is the Kullback–Leibler (KL) divergence of the distribution of *X* and the uniform *k*-mer distribution *U*. It is given by

$$D_{KL}(X||U)=\sum_{i=1}^{4^{k}}v(x_{i})\;\text{log}\;\frac{v(x_{i})}{u(x_{i})}$$

For instance, if k=3 then $u(x_{i})=1/4^{3}=1/64$ constantly, since there exist $4^{3}$ types of 3-mers and each type has the same chance of being selected. A lower KL divergence implies that *X* is more uniformly distributed, and thus the scheme is less biased to specific minimizers. As expected, the results reveal that refined minimizers have lower KL divergence. Therefore, we would expect refined minimizers to generate less biased *k*-mers.

The column *E*-hits is the expected number of hits introduced by research (Sahlin 2022). A lower *E*-hits may benefit applications such as read mapping. It is computed as follows in the assessment.

$$\begin{array}{c} E\;-\;\mathrm{hits}(X)=\frac{1}{\left|X\right|}\sum_{i=1}^{4^{k}}n{\left(x_{i}\right)}^{2}=\frac{1}{\rho |S|}\sum_{i=1}^{4^{k}}n{\left(x_{i}\right)}^{2}\\ =\frac{\left|S\right|}{\rho }\sum_{i=1}^{4^{k}}v{\left(x_{i}\right)}^{2} \end{array}$$

Therefore, it is a comprehensive metric of density ρ and frequency $v(x_{i})$. Since the refined minimizers improve the *k*-mer frequency *V* at the cost of limited increased density ρ, we expect refined minimizers to improve *E*-hits, while the improvement is relatively lower than those of percentiles and $D_{KL}$. *E*-hits for minimizers in GRCH38 are in line with expectations, as shown in Table 3 and Supplementary Tables S1–S3.

**Figure 1.**
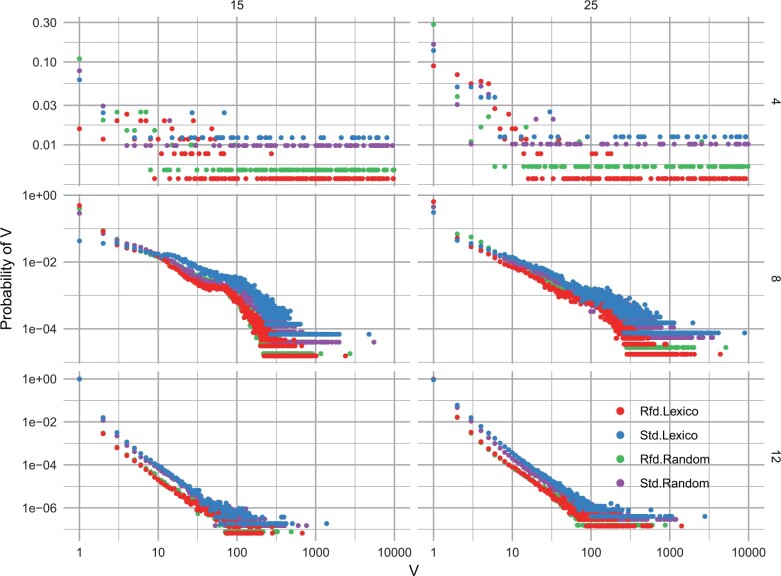
Empirical distributions of *V* for k=4,8,12 in rows and |s|=15,25 in columns. Rfd and Std are refined and standard minimizer. The vertical axis equals the frequency of V=v, namely the empirical probability P(V=v). The horizontal and vertical axes are in ${\;\text{log}\;}_{10}$ scale.

Figure 1 illustrates the empirical distribution of minimizer frequency *V* discussed above. It is log-scaled since the distribution is right-skewed, namely a long tail on the right side. Supplementary Fig. S1 shows the histogram version of the same data as a complement. As discussed above, we prefer small *V* for anchoring and binning problems, since large ones in the long tails would be the performance bottleneck. The figure reveals that for different |s|,k, standard minimizers have heavier tails, indicating larger *V* than refined minimizers. Therefore, refined minimizers generate more uniformly distributed *k*-mers. Figures for additional settings of |s| ⩽ 45 and k ⩽ 30 are presented in Supplementary Fig. S2.

Overall, statistics including the percentiles, $D_{KL}$, *E*-hits and the distribution figures suggest refined lexicographical minimizers are less repetitive than standard lexicographical or random minimizers. Since the refined minimizer is also computationally efficient, it is expected to be more friendly to high-performance minimizer applications.

## 4 Discussion

### 4.1 Potential limitations

We can observe a drop in benefits for frequency-related statistics of refined minimizers for larger *k* and |*s*| (i.e. $P_{0.95}$, $D_{KL}$, *E*-hits, and distributions in Supplementary Fig. S2). However, it is worth noting that the benefits depend on a latent factor, the sequence size. We use a coefficient, the average minimizer occurrences in the sequence denoted by E(X,k) to describe the latent performance impact.

$$E(X,k)=\frac{\left|X\right|}{4^{k}}=\frac{\rho |S|}{4^{k}}\approx \frac{2|S|}{(1+w)4^{k}}=\frac{2|S|}{(|s|-k+2)4^{k}}$$

where ρ≈2/(1+w) is the expected minimizer density. For instance, if we assess 20-mers in GRCH38 references of approximately 3*Gbps* in size, then $E(X,k)=\rho \cdot 3G\mathrm{bps}/4^{20}\approx 0$. It means that most types of 20-mers never occur in the minimizer set of GRCH38. As a result, the empirical distribution of minimizer frequency will not be close to the expected one due to insufficient minimizers (i.e. law of large numbers). Specifically, E(X,k) drops exponentially or linearly as *k* or |*s*| increases. Therefore, given the sequence of fixed size (e.g. GRCH38), we expect to observe significant or moderate drops in the statistics for large *k* or |*s*|. For validation, we assessed the empirical distributions of minimizer frequency *V* for |s|=25,k=10 in 6 sequences, whose sizes |*S*| are 1,4,16,64,256,1024Mbps, as shown in Supplementary Fig. S3. We can observe that the difference between the standard and refined minimizer distributions is insignificant in short sequences (e.g. 1Mbps,4Mbps). However, distributions become significantly different as the sequence size |*S*| increases exponentially. Therefore, the empirical distributions depend on the sequence size and the practical benefits will increase as the sequence size grows.

### 4.2 Potential improvements

We have discussed the heuristic to improve the refined minimizer density in Section 2.3. There potentially exist other heuristics that can improve the refined minimizers in practice. For instance, refined minimizers can possibly be improved for specific sequences, such as *A*, *T* or *C*, *G* enriched ones, where δ signs are likely to be frequently changed. A potential improvement is to extend δ as follows:

$$\delta_{\omega }(s)=\omega_{1}(p_{A}-p_{T})+\omega_{2}(p_{C}-p_{G})$$

where weights $\omega_{1},\omega_{2}\equiv 1\;\left(\mathrm{mod}2\right)$. Additionally, we extend δ based on the occurrences of 2-mers $p_{AA},p_{AC},\ldots ,p_{TT}$ or *q*-mers (i.e. *q* characters). Generally, δ based on the occurrences of *q*-mers can be defined as

$$\delta_{\omega ,q}(s)=\sum_{i=1}^{4^{q}/2}\omega_{i}(p_{q_{i}}-p_{q_{i}^{\prime }})$$

where $q_{i},q_{i}^{\prime }$ are the *i*th *q*-mer and its reverse complement. Weights $\omega_{i}$ can be optimized, provided distributions of *q*-mers in the sequences are known. In practice, the distributions can be approximated by sampling *q*-mers in the subsequences. Such heuristics may further improve the performance of refined minimizers.

## 5 Conclusion

In this work, we proposed a refined DNA minimizer operator. We discussed basic properties that are essential to applications. The refined minimize is generic, computationally efficient, and can improve the *k*-mer repetitiveness, especially for the lexicographic order at the cost of limited increased density. However, simple heuristics, such as skipping “solo” windows, can further improve the performance. Assessments based on the GRCH38 are in line with expectations. We expect the performance can be potentially improved with additional heuristics in practice.

## Supplementary Material

btae045_Supplementary_Data

## Data Availability

The benchmark used in this work is available at https://github.com/xp3i4/mini_benchmark. The data underlying this article are available in the article and in its online supplementary material.
