# Supplementary material for: A simple refined DNA minimizer operator enables 2-fold faster computation
Source: Bioinformatics. 2024 Jan 25;40(2):btae045. doi: 10.1093/bioinformatics/btae045 (PMC10868324; doi:10.1093/bioinformatics/btae045)
Supplement: btae045_Supplementary_Data [file btae045_supplementary_data.pdf]

A simple refined DNA minimizer operator enables twofold faster computation

Chenxu Pan[1] and Knut Reinert[1,2]

[1]*Department of Mathematics and Computer Science, Free University of Berlin, Takustr. 9, 14195 Berlin, Germany*
[2]*Department of Computational Molecular Biology, Max Planck Institute for Molecular Genetics, Berlin 14195, Germany*

Contents

1 Supplementary Tables **2**
1.1 Table of additional settings of $|s|$ and k for lexicographical ordering 2
1.2 Table of additional settings of $|s|$ and k for random ordering 3
1.3 Table of additional settings of $|s|$ and k for minimap2 4

2 Supplementary Figures **5**
2.1 Figure of minimizer frequency distributions using histograms 5
2.2 Figure of minimizer frequency distributions for additional settings of $|s|$ and k 6
2.3 Figure of minimizer frequency distributions for different sequence sizes with $|s| = 25, k = 10$ . . 7

3 Supplementary Notes **8**
3.1 Assessment protocol 8
3.2 Compute minimizers for consecutive windows by using buffers 9

1 Supplementary Tables

1.1 Table of additional settings of $|s|$ and k for lexicographical ordering

$\lvert s\rvert, k$	$P_{0.25}(V)$		$P_{0.5}(V)$		$P_{0.75}(V)$		$P_{0.95}(V)$		$\rho_{<,X}$		D_{KL}		E-hits		T [s]	
	Std	Rfd	Std	Rfd	Std	Rfd	Std	Rfd	Std	Rfd	Std	Rfd	Std	Rfd	Std	Rfd
15,4	9.97	**7.55**	201.92	**69.07**	2648.28	**762.28**	10056.20	**4862.38**	**0.16**	0.21	2.28	**1.26**	2.53E7	**1.14E7**	27.60	**26.12**
15,6	6.00	**0.66**	48.69	**3.80**	274.26	**38.69**	767.05	**371.45**	**0.20**	0.25	2.30	**1.41**	2.16E6	**1.09E6**	27.62	**26.27**
15,8	2.32	**0.06**	7.54	**0.40**	23.66	**2.58**	61.41	**25.48**	**0.26**	0.29	2.25	**1.59**	1.92E5	**1.23E5**	28.88	**26.62**
15,10	0.10	**0.01**	0.39	**0.05**	1.44	**0.21**	4.27	**1.84**	**0.32**	0.35	2.34	**1.74**	3.17E4	**2.11E4**	30.02	**25.63**
15,12	0.01	**0.00**	0.02	**0.01**	0.09	**0.03**	0.28	**0.16**	**0.44**	0.46	2.43	**1.90**	1.29E4	**9.11E3**	31.03	**26.22**
25,4	**0.28**	0.71	22.90	**4.34**	697.16	**170.95**	8593.13	**3880.41**	**0.09**	0.13	2.76	**1.73**	2.43E7	**1.19E7**	25.04	**22.73**
25,6	0.07	**0.04**	6.25	**0.25**	76.73	**8.97**	581.48	**245.37**	**0.11**	0.15	2.88	**1.86**	2.34E6	**1.01E6**	24.89	**22.64**
25,8	0.04	**0.00**	1.04	**0.02**	7.27	**0.69**	44.77	**16.95**	**0.12**	0.16	3.01	**2.06**	2.26E5	**9.93E4**	25.03	**22.63**
25,10	0.02	**0.00**	0.13	**0.01**	0.67	**0.09**	3.30	**1.32**	**0.14**	0.17	3.17	**2.33**	3.58E4	**1.83E4**	27.29	**22.64**
25,12	0.00	**0.00**	0.01	**0.00**	0.06	**0.01**	0.24	**0.12**	**0.16**	0.19	3.40	**2.67**	1.34E4	**8.32E3**	25.85	**23.02**
25,14	0.00	**0.00**	0.00	**0.00**	0.01	**0.00**	0.02	**0.01**	**0.19**	0.21	3.60	**2.99**	7.01E3	**5.61E3**	26.97	**23.62**
25,16	0.00	**0.00**	0.00	**0.00**	0.00	**0.00**	0.00	**0.00**	**0.21**	0.24	4.09	**3.68**	4.48E3	**3.56E3**	27.58	**24.47**
25,18	0.00	**0.00**	0.00	**0.00**	0.00	**0.00**	0.00	**0.00**	**0.26**	0.28	5.60	**5.44**	2.91E3	**2.49E3**	27.35	**25.03**
25,20	0.00	**0.00**	0.00	**0.00**	0.00	**0.00**	0.00	**0.00**	**0.33**	0.35	7.86	**7.76**	1.86E3	**1.61E3**	28.67	**24.21**
25,22	0.00	**0.00**	0.00	**0.00**	0.00	**0.00**	0.00	**0.00**	**0.44**	0.46	10.20	**10.14**	1.37E3	**1.17E3**	30.93	**25.04**
35,4	**0.06**	0.11	6.89	**1.06**	303.57	**37.75**	7.36E3	**3.15E3**	**0.06**	0.10	3.07	**2.06**	2.33E7	**1.25E7**	24.91	**21.44**
35,6	0.01	**0.01**	1.33	**0.05**	36.18	**2.52**	457.67	**187.34**	**0.08**	0.11	3.26	**2.21**	2.5E6	**1.1E6**	23.47	**20.89**
35,8	0.02	**0.00**	0.37	**0.01**	3.85	**0.36**	37.62	**13.76**	**0.08**	0.11	3.43	**2.40**	2.54E5	**1.03E5**	23.42	**21.00**
35,10	0.01	**0.00**	0.06	**0.01**	0.36	**0.09**	2.78	**1.26**	**0.09**	0.12	3.65	**2.66**	4.32E4	**1.69E4**	23.65	**21.21**
35,12	0.00	**0.00**	0.01	**0.00**	0.03	**0.01**	0.20	**0.12**	**0.10**	0.13	3.96	**3.00**	1.65E4	**7.39E3**	23.44	**21.40**
35,14	0.00	**0.00**	0.00	**0.00**	0.00	**0.00**	0.02	**0.01**	**0.10**	0.13	4.23	**3.37**	8.3E3	**4.9E3**	23.74	**21.62**
35,16	0.00	**0.00**	0.00	**0.00**	0.00	**0.00**	0.00	**0.00**	**0.12**	0.14	4.72	**4.16**	5E3	**3.46E3**	24.33	**21.82**
35,18	0.00	**0.00**	0.00	**0.00**	0.00	**0.00**	0.00	**0.00**	**0.13**	0.15	6.29	**6.04**	3.18E3	**2.5E3**	25.20	**21.89**
35,20	0.00	**0.00**	0.00	**0.00**	0.00	**0.00**	0.00	**0.00**	**0.14**	0.17	8.65	**8.49**	1.85E3	**1.65E3**	25.10	**21.96**
35,22	0.00	**0.00**	0.00	**0.00**	0.00	**0.00**	0.00	**0.00**	**0.16**	0.18	11.19	**11.05**	1.15E3	**914**	25.76	**22.30**
35,24	0.00	**0.00**	0.00	**0.00**	0.00	**0.00**	0.00	**0.00**	**0.18**	0.21	13.75	**13.63**	746	**653**	27.25	**22.92**
35,26	0.00	**0.00**	0.00	**0.00**	0.00	**0.00**	0.00	**0.00**	**0.21**	0.24	16.30	**16.21**	554	**500**	27.53	**23.76**
35,28	0.00	**0.00**	0.00	**0.00**	0.00	**0.00**	0.00	**0.00**	**0.26**	0.28	18.84	**18.77**	420	**374**	27.35	**24.49**
35,30	0.00	**0.00**	0.00	**0.00**	0.00	**0.00**	0.00	**0.00**	**0.32**	0.34	21.33	**21.28**	310	**284**	28.68	**23.73**
45,4	**0.01**	0.03	2.04	**0.35**	130.88	**13.58**	6316.96	**2316.08**	**0.05**	0.08	3.31	**2.32**	2.23E7	**1.28E7**	25.11	**20.88**
45,6	0.01	**0.00**	0.72	**0.03**	22.36	**1.29**	393.57	**153.16**	**0.06**	0.09	3.54	**2.48**	2.62E6	**1.19E6**	22.82	**15.68**
45,8	0.01	**0.00**	0.20	**0.01**	2.43	**0.38**	31.31	**12.85**	**0.06**	0.09	3.74	**2.67**	2.79E5	**1.07E5**	22.68	**15.70**
45,10	0.00	**0.00**	0.04	**0.01**	0.24	**0.09**	2.39	**1.24**	**0.07**	0.09	3.99	**2.92**	4.98E4	**1.61E4**	22.72	**15.72**
45,12	0.00	**0.00**	0.01	**0.00**	0.03	**0.02**	0.19	**0.12**	**0.07**	0.10	4.32	**3.26**	1.91E4	**7.05E3**	25.04	**15.79**
45,14	0.00	**0.00**	0.00	**0.00**	0.00	**0.00**	0.02	**0.01**	**0.07**	0.10	4.63	**3.63**	9.69E3	**4.6E3**	23.79	**15.87**
45,16	0.00	**0.00**	0.00	**0.00**	0.00	**0.00**	0.00	**0.00**	**0.08**	0.10	5.17	**4.44**	5.4E3	**3.18E3**	23.49	**15.96**
45,18	0.00	**0.00**	0.00	**0.00**	0.00	**0.00**	0.00	**0.00**	**0.09**	0.11	6.76	**6.37**	3.31E3	**2.4E3**	23.43	**16.04**
45,20	0.00	**0.00**	0.00	**0.00**	0.00	**0.00**	0.00	**0.00**	**0.09**	0.12	9.13	**8.87**	1.98E3	**1.67E3**	23.74	**16.18**
45,22	0.00	**0.00**	0.00	**0.00**	0.00	**0.00**	0.00	**0.00**	**0.10**	0.12	11.67	**11.48**	1.21E3	**1.06E3**	23.65	**16.80**
45,24	0.00	**0.00**	0.00	**0.00**	0.00	**0.00**	0.00	**0.00**	**0.11**	0.13	14.25	**14.12**	753	**678**	24.43	**16.41**
45,26	0.00	**0.00**	0.00	**0.00**	0.00	**0.00**	0.00	**0.00**	**0.12**	0.14	16.89	**16.76**	531	**474**	24.31	**16.70**
45,28	0.00	**0.00**	0.00	**0.00**	0.00	**0.00**	0.00	**0.00**	**0.13**	0.15	19.51	**19.39**	367	**316**	24.86	**16.79**
45,30	0.00	**0.00**	0.00	**0.00**	0.00	**0.00**	0.00	**0.00**	**0.14**	0.16	22.13	**22.03**	240	**232**	24.98	**16.67**

1.2 Table of additional settings of $|s|$ and k for random ordering

$\lvert s\rvert, k$	$P_{0.25}(V)$		$P_{0.5}(V)$		$P_{0.75}(V)$		$P_{0.95}(V)$		$\rho_{<,X}$		D_{KL}		E-hits		T [s]	
	Std	Rfd	Std	Rfd	Std	Rfd	Std	Rfd	Std	Rfd	Std	Rfd	Std	Rfd	Std	Rfd
15,4	7.82	**1.56**	163.57	**88.59**	1738.06	**1273.00**	7921.35	**4804.52**	**0.15**	0.19	2.14	**1.51**	2.26E7	**1.59E7**	48.57	**42.94**
15,6	0.97	**0.33**	14.43	**6.90**	126.35	**58.87**	564.40	**386.08**	**0.18**	0.22	2.11	**1.58**	1.62E6	**1.24E6**	48.31	**43.24**
15,8	0.14	**0.04**	1.33	**0.48**	8.75	**3.70**	41.05	**25.83**	**0.22**	0.25	2.17	**1.67**	1.6E5	**1.26E5**	47.95	**43.21**
15,10	0.05	**0.01**	0.22	**0.05**	1.02	**0.30**	3.35	**2.01**	**0.30**	0.33	2.21	**1.84**	2.98E4	**2.56E4**	50.19	**42.23**
15,12	0.00	**0.00**	0.01	**0.01**	0.06	**0.04**	0.23	**0.17**	**0.41**	0.42	2.31	**2.06**	1.07E4	**1.02E4**	52.04	**41.94**
25,4	0.34	**0.07**	**6.32**	6.40	634.58	**472.81**	5710.84	**3480.47**	**0.09**	0.12	2.66	**2.01**	2.25E7	**1.71E7**	46.20	**38.61**
25,6	0.03	**0.03**	1.81	**1.52**	45.23	**27.07**	439.63	**310.45**	**0.10**	0.13	2.71	**2.14**	1.69E6	**1.32E6**	45.73	**38.56**
25,8	0.01	**0.01**	0.24	**0.19**	3.62	**2.03**	31.74	**22.66**	**0.11**	0.14	2.83	**2.29**	1.61E5	**1.34E5**	45.89	**38.23**
25,10	0.01	**0.00**	0.06	**0.03**	0.38	**0.19**	2.31	**1.56**	**0.13**	0.16	2.91	**2.42**	3.04E4	**2.61E4**	45.86	**39.37**
25,12	0.00	**0.00**	0.01	**0.00**	0.03	**0.02**	0.17	**0.12**	**0.14**	0.17	3.18	**2.74**	**8.97E3**	9.64E3	46.79	**41.85**
25,14	0.00	**0.00**	0.00	**0.00**	0.00	**0.00**	0.01	**0.01**	**0.16**	0.18	3.46	**3.04**	6.1E3	**5.99E3**	47.40	**39.40**
25,16	0.00	**0.00**	0.00	**0.00**	0.00	**0.00**	0.00	**0.00**	**0.18**	0.21	4.02	**3.74**	**3.57E3**	3.61E3	48.09	**39.54**
25,18	0.00	**0.00**	0.00	**0.00**	0.00	**0.00**	0.00	**0.00**	**0.22**	0.24	5.67	**5.54**	**2.48E3**	2.6E3	48.13	**40.51**
25,20	0.00	**0.00**	0.00	**0.00**	0.00	**0.00**	0.00	**0.00**	**0.29**	0.30	7.95	**7.88**	1.75E3	**1.73E3**	49.48	**39.77**
25,22	0.00	**0.00**	0.00	**0.00**	0.00	**0.00**	0.00	**0.00**	**0.40**	0.41	10.28	**10.25**	1.27E3	**1.21E3**	53.39	**40.16**
35,4	0.12	**0.02**	2.01	**0.93**	245.40	**211.28**	3813.66	**3375.80**	**0.06**	0.08	3.03	**2.37**	2.24E7	**1.8E7**	47.34	**37.08**
35,6	0.01	**0.01**	0.43	**0.27**	20.07	**13.85**	365.99	**286.10**	**0.07**	0.09	3.10	**2.50**	1.76E6	**1.43E6**	44.43	**36.49**
35,8	0.01	**0.00**	0.23	**0.06**	3.42	**1.61**	29.57	**21.24**	**0.07**	0.10	3.22	**2.67**	1.74E5	**1.45E5**	44.40	**36.08**
35,10	0.00	**0.00**	0.04	**0.02**	0.29	**0.18**	2.07	**1.51**	**0.08**	0.11	3.31	**2.79**	3.4E4	**2.87E4**	44.73	**36.43**
35,12	0.00	**0.00**	0.01	**0.00**	0.03	**0.02**	0.15	**0.12**	**0.08**	0.11	3.64	**3.13**	9.27E3	**9.07E3**	44.82	**36.70**
35,14	0.00	**0.00**	0.00	**0.00**	0.00	**0.00**	0.01	**0.01**	**0.09**	0.12	3.98	**3.48**	6.31E3	**4.92E3**	45.09	**36.80**
35,16	0.00	**0.00**	0.00	**0.00**	0.00	**0.00**	0.00	**0.00**	**0.10**	0.12	4.60	**4.26**	**3.12E3**	3.67E3	45.67	**36.88**
35,18	0.00	**0.00**	0.00	**0.00**	0.00	**0.00**	0.00	**0.00**	**0.11**	0.13	6.39	**6.18**	**2.32E3**	2.38E3	45.98	**37.30**
35,20	0.00	**0.00**	0.00	**0.00**	0.00	**0.00**	0.00	**0.00**	**0.12**	0.14	8.81	**8.64**	1.28E3	**1.28E3**	46.04	**38.32**
35,22	0.00	**0.00**	0.00	**0.00**	0.00	**0.00**	0.00	**0.00**	**0.13**	0.16	11.35	**11.21**	1.12E3	**946**	46.69	**38.05**
35,24	0.00	**0.00**	0.00	**0.00**	0.00	**0.00**	0.00	**0.00**	**0.15**	0.18	13.91	**13.78**	717	**681**	47.35	**37.98**
35,26	0.00	**0.00**	0.00	**0.00**	0.00	**0.00**	0.00	**0.00**	**0.18**	0.20	16.46	**16.36**	595	**560**	48.12	**38.43**
35,28	0.00	**0.00**	0.00	**0.00**	0.00	**0.00**	0.00	**0.00**	**0.22**	0.24	18.98	**18.90**	392	**385**	48.24	**38.93**
35,30	0.00	**0.00**	0.00	**0.00**	0.00	**0.00**	0.00	**0.00**	**0.29**	0.30	21.46	**21.41**	308	**294**	49.63	**39.49**
45,4	0.06	**0.01**	0.57	**0.36**	**64.66**	72.24	2692.24	**2647.85**	**0.04**	0.07	3.31	**2.64**	2.2E7	**1.85E7**	45.43	**37.75**
45,6	0.00	**0.00**	0.09	**0.07**	7.43	**6.40**	300.07	**244.27**	**0.05**	0.08	3.38	**2.77**	1.84E6	**1.53E6**	43.66	**37.28**
45,8	0.00	**0.00**	0.12	**0.04**	2.36	**1.35**	27.03	**21.12**	**0.05**	0.08	3.51	**2.94**	1.88E5	**1.57E5**	45.07	**35.35**
45,10	0.00	**0.00**	0.03	**0.03**	0.23	**0.19**	1.91	**1.58**	**0.06**	0.09	3.62	**3.06**	3.9E4	**3.12E4**	43.46	**35.38**
45,12	0.00	**0.00**	0.00	**0.00**	0.02	**0.02**	0.15	**0.12**	**0.06**	0.08	3.95	**3.42**	**9.45E3**	9.64E3	43.80	**35.04**
45,14	0.00	**0.00**	0.00	**0.00**	0.00	**0.00**	0.01	**0.01**	**0.06**	0.09	4.30	**3.79**	6.35E3	**4.86E3**	44.04	**34.92**
45,16	0.00	**0.00**	0.00	**0.00**	0.00	**0.00**	0.00	**0.00**	**0.07**	0.09	4.97	**4.60**	**2.76E3**	3.6E3	43.58	**34.99**
45,18	0.00	**0.00**	0.00	**0.00**	0.00	**0.00**	0.00	**0.00**	**0.07**	0.09	6.80	**6.55**	**2.25E3**	2.34E3	44.38	**35.54**
45,20	0.00	**0.00**	0.00	**0.00**	0.00	**0.00**	0.00	**0.00**	**0.07**	0.10	9.26	**9.05**	1.22E3	**1.18E3**	44.75	**35.50**
45,22	0.00	**0.00**	0.00	**0.00**	0.00	**0.00**	0.00	**0.00**	**0.08**	0.10	11.86	**11.66**	1.05E3	**840**	47.47	**35.26**
45,24	0.00	**0.00**	0.00	**0.00**	0.00	**0.00**	0.00	**0.00**	**0.09**	0.11	14.47	**14.29**	**634**	660	46.04	**35.50**
45,26	0.00	**0.00**	0.00	**0.00**	0.00	**0.00**	0.00	**0.00**	**0.10**	0.12	17.09	**16.93**	523	**519**	45.74	**35.83**
45,28	0.00	**0.00**	0.00	**0.00**	0.00	**0.00**	0.00	**0.00**	**0.11**	0.13	19.71	**19.56**	348	**342**	47.51	**35.85**
45,30	0.00	**0.00**	0.00	**0.00**	0.00	**0.00**	0.00	**0.00**	**0.12**	0.14	22.33	**22.20**	270	**261**	46.26	**37.15**

1.3 Table of additional settings of $|s|$ and k for minimap2

Minimizers used by minimap2 (mm2, $k \leqslant 28$ allowed) is compared to standard random minimizers (Std) and refined lexicographic minimizers(Rfd). Since standard minimizers perform better when using random order. Refined minimizers perform better when using lexicographic order.

$\|s\|, k$	$P_{0.25}(V)$			$P_{0.5}(V)$			$P_{0.75}(V)$			$P_{0.95}(V)$			$\rho_{<,X}$			D_{KL}			E-hits			T [s]		
	mm2	Std	Rfd	mm2	Std	Rfd	mm2	Std	Rfd	mm2	Std	Rfd	mm2	Std	Rfd	mm2	Std	Rfd	mm2	Std	Rfd	mm2	Std	Rfd
15,4	**4.62**	7.82	7.55	97.06	163.57	**69.07**	1927.42	1738.06	**762.28**	8320.94	7921.35	**4862.38**	0.16	**0.15**	0.21	2.20	2.14	**1.26**	2.2E7	2.3E7	**1.1E7**	40.34	48.57	**26.12**
15,6	0.86	0.97	**0.66**	17.27	14.43	**3.80**	123.08	126.35	**38.69**	587.75	564.40	**371.45**	0.18	**0.18**	0.25	2.13	2.11	**1.41**	1.6E6	1.6E6	**1.1E6**	42.01	48.31	**26.27**
15,8	0.14	0.14	**0.06**	1.34	1.33	**0.40**	8.76	8.75	**2.58**	40.80	41.05	**25.48**	0.22	**0.22**	0.29	2.19	2.17	**1.59**	1.9E5	1.6E5	**1.2E5**	41.41	47.95	**26.62**
15,10	0.03	0.05	**0.01**	0.16	0.22	**0.05**	0.80	1.02	**0.21**	3.13	3.35	**1.84**	0.30	**0.30**	0.35	2.24	2.21	**1.74**	4.9E4	3E4	**2.1E4**	43.85	50.19	**25.63**
15,12	0.00	0.00	**0.00**	0.01	0.01	**0.01**	0.06	0.06	**0.03**	0.22	0.23	**0.16**	0.41	**0.41**	0.46	2.33	2.31	**1.90**	2.2E4	1.1E4	**9.1E3**	45.78	52.04	**26.22**
25,4	**0.13**	0.34	0.71	19.88	6.32	**4.34**	922.62	634.58	**170.95**	5583.97	5710.84	**3880.41**	0.09	**0.09**	0.13	2.70	2.66	**1.73**	2.3E7	2.2E7	**1.2E7**	35.08	46.20	**22.73**
25,6	0.05	**0.03**	0.04	3.21	1.81	**0.25**	49.59	45.23	**8.97**	447.86	439.63	**245.37**	0.10	**0.10**	0.15	2.70	2.71	**1.86**	1.5E6	1.7E6	**1E6**	36.76	45.73	**22.64**
25,8	0.01	0.01	**0.00**	0.31	0.24	**0.02**	3.28	3.62	**0.69**	32.09	31.74	**16.95**	**0.10**	0.11	0.16	2.86	2.83	**2.06**	1.5E5	1.6E5	**9.9E4**	37.60	45.89	**22.63**
25,10	0.01	0.01	**0.00**	0.05	0.06	**0.01**	0.32	0.38	**0.09**	2.28	2.31	**1.32**	**0.12**	0.13	0.17	2.96	2.91	**2.33**	2.7E4	3E4	**1.8E4**	38.62	45.86	**22.64**
25,12	0.00	0.00	**0.00**	0.01	0.01	**0.00**	0.03	0.03	**0.01**	0.17	0.17	**0.12**	0.14	**0.14**	0.19	3.22	3.18	**2.67**	1.1E4	9E3	**8.3E3**	39.65	46.79	**23.02**
25,14	0.00	0.00	**0.00**	0.00	0.00	**0.00**	0.00	0.00	**0.00**	0.01	0.01	**0.01**	0.16	**0.16**	0.21	3.48	3.46	**2.99**	6.9E3	6.1E3	**5.6E3**	41.56	47.40	**23.62**
25,16	0.00	0.00	**0.00**	0.00	0.00	**0.00**	0.00	0.00	**0.00**	0.00	0.00	**0.00**	0.18	**0.18**	0.24	4.02	4.02	**3.68**	4.1E3	3.6E3	**3.6E3**	43.31	48.09	**24.47**
25,18	0.00	0.00	**0.00**	0.00	0.00	**0.00**	0.00	0.00	**0.00**	0.00	0.00	**0.00**	0.22	**0.22**	0.28	5.68	5.67	**5.44**	3.8E3	2.5E3	**2.5E3**	41.87	48.13	**25.03**
25,20	0.00	0.00	**0.00**	0.00	0.00	**0.00**	0.00	0.00	**0.00**	0.00	0.00	**0.00**	0.29	**0.29**	0.35	7.96	7.95	**7.76**	2.3E3	1.8E3	**1.6E3**	43.87	49.48	**24.21**
25,22	0.00	0.00	**0.00**	0.00	0.00	**0.00**	0.00	0.00	**0.00**	0.00	0.00	**0.00**	0.40	**0.40**	0.46	10.28	10.28	**10.14**	1.6E3	1.3E3	**1.2E3**	45.44	53.39	**25.04**
35,4	**0.05**	0.12	0.11	5.23	2.01	**1.06**	548.38	245.40	**37.75**	6753.06	3813.66	**3150.43**	0.07	**0.06**	0.10	3.05	3.03	**2.06**	2.4E7	2.2E7	**1.2E7**	32.10	47.34	**21.44**
35,6	0.01	0.01	**0.01**	0.61	0.43	**0.05**	23.85	20.07	**2.52**	403.56	365.99	**187.34**	0.07	**0.07**	0.11	3.08	3.10	**2.21**	1.6E6	1.8E6	**1.1E6**	33.59	44.43	**20.89**
35,8	0.01	0.01	**0.00**	0.09	0.23	**0.01**	2.28	3.42	**0.36**	28.51	29.57	**13.76**	0.07	**0.07**	0.11	3.25	3.22	**2.40**	1.4E5	1.7E5	**1E5**	34.07	44.40	**21.00**
35,10	0.00	0.00	**0.00**	0.03	0.04	**0.01**	0.24	0.29	**0.09**	2.02	2.07	**1.26**	0.08	**0.08**	0.12	3.35	3.31	**2.66**	2.1E4	3.4E4	**1.7E4**	35.08	44.73	**21.21**
35,12	0.00	0.00	**0.00**	0.01	0.01	**0.00**	0.03	0.03	**0.01**	0.16	0.15	**0.12**	0.08	**0.08**	0.13	3.68	3.64	**3.00**	1.1E4	9.3E3	**7.4E3**	35.79	44.82	**21.40**
35,14	0.00	0.00	**0.00**	0.00	0.00	**0.00**	0.00	0.00	**0.00**	0.01	0.01	**0.01**	0.09	**0.09**	0.13	4.00	3.98	**3.37**	7.6E3	6.3E3	**4.9E3**	37.52	45.09	**21.62**
35,16	0.00	0.00	**0.00**	0.00	0.00	**0.00**	0.00	0.00	**0.00**	0.00	0.00	**0.00**	0.10	**0.10**	0.14	4.62	4.60	**4.16**	3.8E3	**3.1E3**	3.5E3	36.95	45.67	**21.82**
35,18	0.00	0.00	**0.00**	0.00	0.00	**0.00**	0.00	0.00	**0.00**	0.00	0.00	**0.00**	0.11	**0.11**	0.15	6.40	6.39	**6.04**	2.8E3	**2.3E3**	2.5E3	37.90	45.98	**21.89**
35,20	0.00	0.00	**0.00**	0.00	0.00	**0.00**	0.00	0.00	**0.00**	0.00	0.00	**0.00**	0.12	**0.12**	0.17	8.82	8.81	**8.49**	1.8E3	**1.3E3**	1.6E3	38.24	46.04	**21.96**
35,22	0.00	0.00	**0.00**	0.00	0.00	**0.00**	0.00	0.00	**0.00**	0.00	0.00	**0.00**	0.13	**0.13**	0.18	11.36	11.35	**11.05**	1.6E3	1.1E3	**9.1E2**	39.44	46.69	**22.30**
35,24	0.00	0.00	**0.00**	0.00	0.00	**0.00**	0.00	0.00	**0.00**	0.00	0.00	**0.00**	0.15	**0.15**	0.21	13.92	13.91	**13.63**	1E3	7.2E2	**6.5E2**	41.30	47.35	**22.92**
35,26	0.00	0.00	**0.00**	0.00	0.00	**0.00**	0.00	0.00	**0.00**	0.00	0.00	**0.00**	0.18	**0.18**	0.24	16.46	16.46	**16.21**	7.1E2	5.9E2	**5E2**	41.86	48.12	**23.76**
35,28	0.00	0.00	**0.00**	0.00	0.00	**0.00**	0.00	0.00	**0.00**	0.00	0.00	**0.00**	0.22	**0.22**	0.28	18.98	18.98	**18.77**	4.9E2	3.9E2	**3.7E2**	41.54	48.24	**24.49**
45,4	**0.02**	0.06	0.03	1.87	0.57	**0.35**	168.91	64.66	**13.58**	5207.21	2692.24	**2316.08**	0.05	**0.04**	0.08	3.31	3.31	**2.32**	2.4E7	2.2E7	**1.3E7**	29.85	45.43	**20.88**
45,6	0.01	0.00	**0.00**	0.17	0.09	**0.03**	14.92	7.43	**1.29**	333.89	300.07	**153.16**	0.05	**0.05**	0.09	3.36	3.38	**2.48**	1.6E6	1.8E6	**1.2E6**	31.23	43.66	**15.68**
45,8	0.00	0.00	**0.00**	0.04	0.12	**0.01**	1.64	2.36	**0.38**	25.69	27.03	**12.85**	0.05	**0.05**	0.09	3.53	3.51	**2.67**	1.4E5	1.9E5	**1.1E5**	31.55	45.07	**15.70**
45,10	0.00	0.00	**0.00**	0.03	0.03	**0.01**	0.22	0.23	**0.09**	1.93	1.91	**1.24**	0.06	**0.06**	0.09	3.63	3.62	**2.92**	2E4	3.9E4	**1.6E4**	32.16	43.46	**15.72**
45,12	0.00	0.00	**0.00**	0.01	0.00	**0.00**	0.02	0.02	**0.02**	0.15	0.15	**0.12**	0.06	**0.06**	0.10	3.99	3.95	**3.26**	1E4	9.5E3	**7E3**	32.57	43.80	**15.79**
45,14	0.00	0.00	**0.00**	0.00	0.00	**0.00**	0.00	0.00	**0.00**	0.01	0.01	**0.01**	0.06	**0.06**	0.10	4.34	4.30	**3.63**	7.9E3	6.3E3	**4.6E3**	33.00	44.04	**15.87**
45,16	0.00	0.00	**0.00**	0.00	0.00	**0.00**	0.00	0.00	**0.00**	0.00	0.00	**0.00**	0.07	**0.07**	0.10	4.98	4.97	**4.44**	3E3	**2.8E3**	3.2E3	33.42	43.58	**15.96**
45,18	0.00	0.00	**0.00**	0.00	0.00	**0.00**	0.00	0.00	**0.00**	0.00	0.00	**0.00**	0.07	**0.07**	0.11	6.81	6.80	**6.37**	2.4E3	**2.3E3**	2.4E3	33.64	44.38	**16.04**
45,20	0.00	0.00	**0.00**	0.00	0.00	**0.00**	0.00	0.00	**0.00**	0.00	0.00	**0.00**	0.07	**0.07**	0.12	9.27	9.26	**8.87**	1.4E3	**1.2E3**	1.7E3	34.22	44.75	**16.18**
45,22	0.00	0.00	**0.00**	0.00	0.00	**0.00**	0.00	0.00	**0.00**	0.00	0.00	**0.00**	0.08	**0.08**	0.12	11.87	11.86	**11.48**	1.8E3	**1E3**	1.1E3	34.82	47.47	**16.80**
45,24	0.00	0.00	**0.00**	0.00	0.00	**0.00**	0.00	0.00	**0.00**	0.00	0.00	**0.00**	0.09	**0.09**	0.13	14.48	14.47	**14.12**	1E3	**6.3E2**	6.8E2	36.10	46.04	**16.41**
45,26	0.00	0.00	**0.00**	0.00	0.00	**0.00**	0.00	0.00	**0.00**	0.00	0.00	**0.00**	0.10	**0.10**	0.14	17.10	17.09	**16.76**	6.2E2	5.2E2	**4.7E2**	36.99	45.74	**16.70**
45,28	0.00	0.00	**0.00**	0.00	0.00	**0.00**	0.00	0.00	**0.00**	0.00	0.00	**0.00**	0.11	**0.11**	0.15	19.72	19.71	**19.39**	4.1E2	3.5E2	**3.2E2**	37.76	47.51	**16.79**

2 Supplementary Figures

2.1 Figure of minimizer frequency distributions using histograms

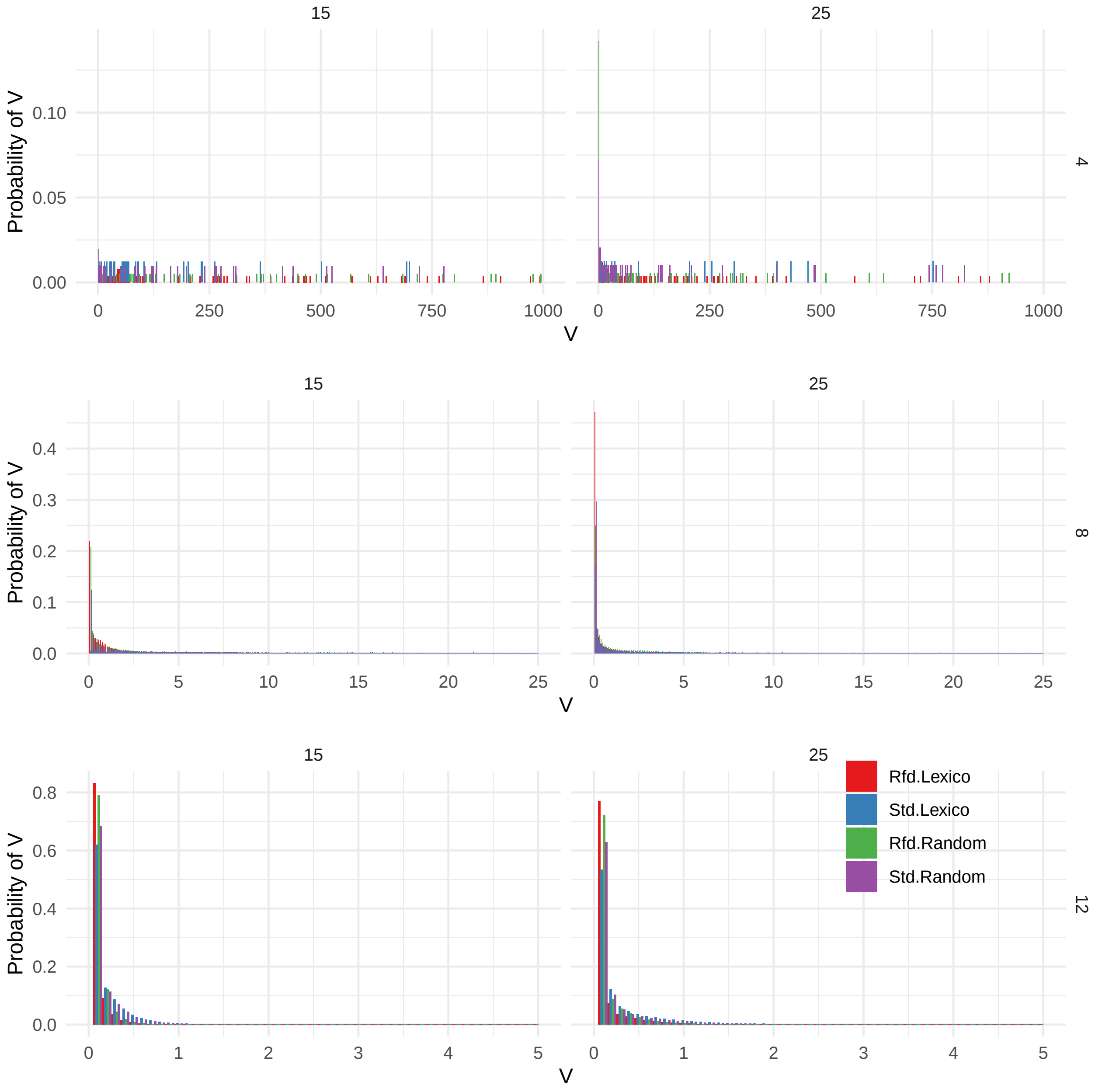

Figure 1: Empirical distributions of V for $k = 4, 8, 12$ in rows and $|s| = 15, 25$ in columns. Rfd and Std are refined and standard minimizers. The figure is the histogram version of the same data used by manuscript Figure 1, whose axes are log-scaled. Histogram bin size $= 0.1$,

2.2 Figure of minimizer frequency distributions for additional settings of $|s|$ and k

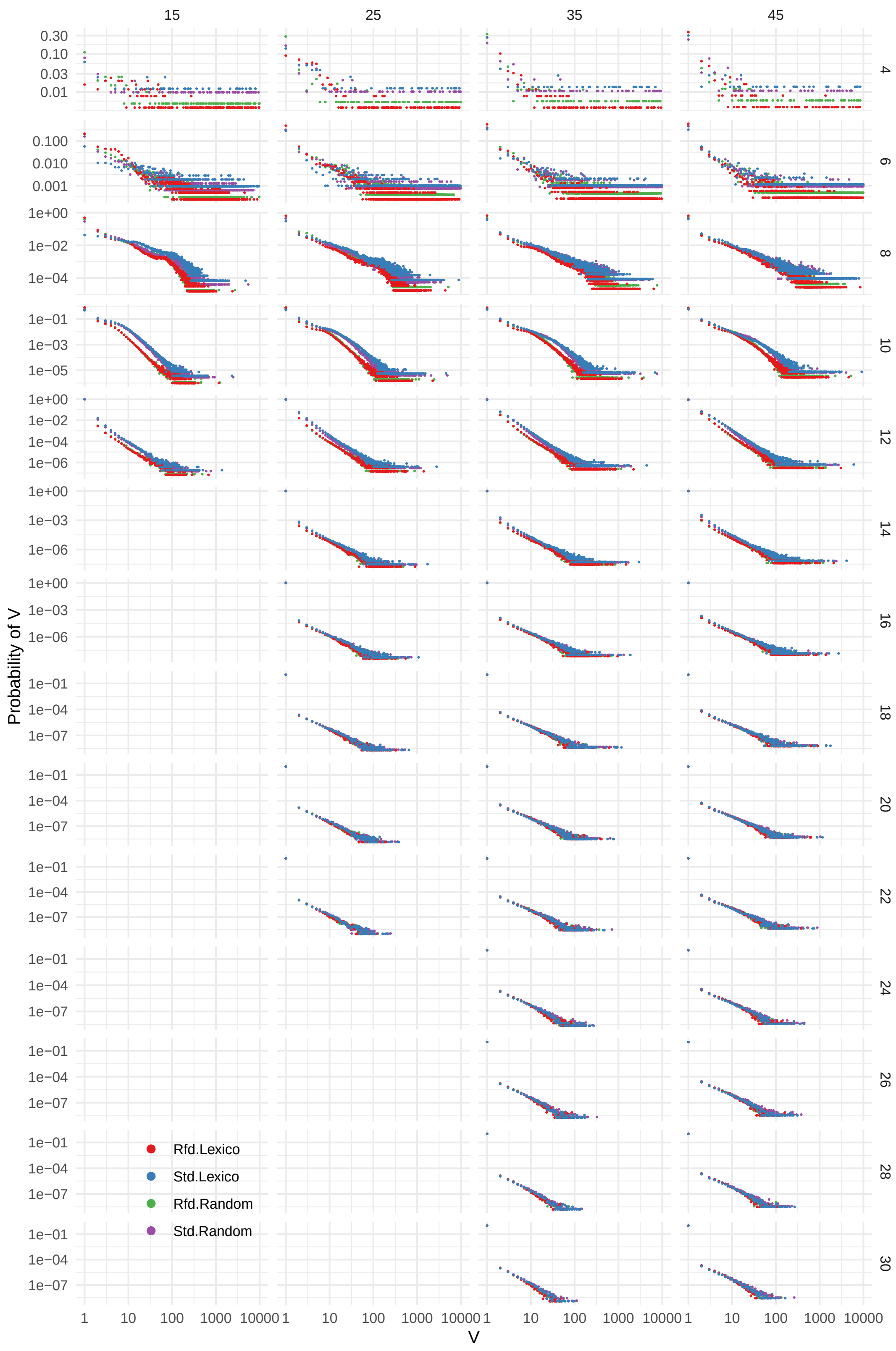

Figure 2: Empirical distributions of V for $k \leqslant 30, |s| \leqslant 45$ in rows and columns. Axes are in $\log_{10}$ scale.

2.3 Figure of minimizer frequency distributions for different sequence sizes with $|s| = 25, k = 10$

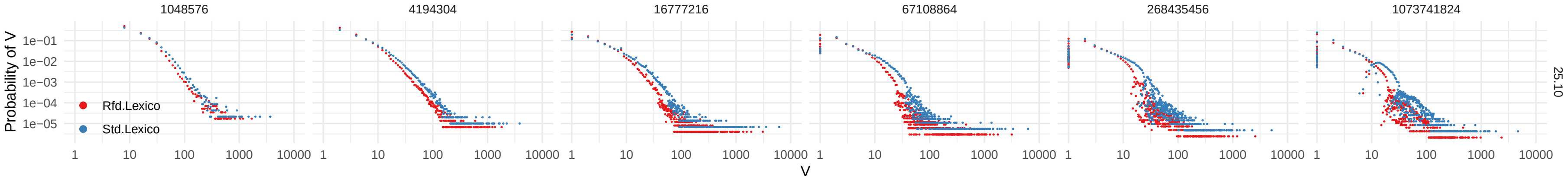

Figure 3: Empirical distributions of V for $|s| = 25, k = 10$ in rows and sequence lengths $= 1M, 4M, 16M, 64M, 256M, 1024M$ in columns. Axes are in $\log_{10}$ scale.

3 Supplementary Notes

3.1 Assessment protocol

Settings of minimap2 The source code is from repository `https://github.com/lh3/minimap2`. The evaluation is based on the source code function mm_sketch function, which is purely for sketching the sequence with minimizers. The function argument is_hpc, which computes homopolymer compressed (HPC) k-mers, is disabled. HPC k-mers remove all consecutive duplicated characters. For example, the HPC k-mer of sequence $GGATTTTCCA$ is $GATCA$. This heuristic is used to improve overlap sensitivity for long-read mapping. Since k of HPC is dynamic, it does not apply to the assessment, which uses fixed $|s|$ and k. Moreover, the heuristics apply to standard and refined minimizers as well in real applications. Therefore, it is disabled in the assessment.

Settings of standard and refined minimizers Figures and tables in the work are based on the benchmark settings as follows: Minimizers are collected consecutively from GRCH38. Implementations are based on Supplementary Notes algorithms 1 and 2. The heuristic discussed in the work is employed to skip solo windows for refined minimizers. The benchmark runs with one thread.

3.2 Compute minimizers for consecutive windows by using buffers

The idea of using buffers for consecutive windows $s_i, i = 1, 2, ..n$ is to record k-mers and minimizers in buffers, and thus they can be fetched directly when iterating consecutive windows s_i. We compared functions of computing standard minimizers denoted by $h_s(s_i)$ and refined minimizers denoted by $h_r(s_i)$ in consecutive windows when using fixed-capacity buffers. Ideally, it is at most 2 times faster to compute refined minimizers, since $h_r(s_i)$ either computes minimizers in the forward sequence when $\delta_i > 0$ (i.e. δ of $s_i > 0$) or the reverse complement when $\delta_i < 0$. Specifically, denote i_k the kth i that $\delta_{i_k-1}\delta_{i_k} < 0$, as shown in Figure 4. Denote $o_r(s_i)$ the time complexity of computing the ith refined minimizer. There exist two cases regarding $o_r(s_i)$,

$$o_r(s_i) = \begin{cases} o_h + o_m & \text{If } i_{k-1} \leqslant i < i_k - |s| + 1 \\ o_h + 2o_m & \text{If } i_k - |s| + 1 \leqslant i < i_k \end{cases}$$

where o_h is the time complexity of headers (i.e. additional buffer operations) and o_m is for computing the order of single-strand k-mer (i.e. hashing and comparing k-mer). For instance, the time complexity of computing refined miniminizers $h_r(s_i)$ in windows sliding from base 6 to base 21 as shown in Figure 4 is $\sum_{i=6}^{i<21} o_r(s_i) = 13(o_h + o_m) + 2(o_h + 2o_m)$. In contrast, the standard minimizer function $h_s(s_i)$ always iterates all k-mers of both forward and reverse windows. Therefore the time complexity $o_s(s_i) = 2o_m$. Thus the speedup T_r is

$$\frac{2o_m}{o_h + 2o_m} \leqslant T_r = \frac{o_s}{o_r} \leqslant \frac{2o_m}{o_h + o_m}$$

When $o_h \ll o_m$, the maximum T_r is 2 times faster at most. Algorithms 1 and 2 show workflows computing the refined and standard minimizers in consecutive windows. Algorithms 1 defines a w-bit integer Δ_i to record the signs of $\delta_i, ..., \delta_{i+w-1}$, whose windows $s_i, ..., s_{i+w-1}$ contain the ith k-mer. Δ_i can be iterated. If all bits of Δ_i equal 0 or 1 (i.e. $\Delta_i = 0$ or $2^w - 1$), then only the forward or reverse ith k-mer is required by $s_i, ..., s_{i+w-1}$. In such case, only the forward or reverse strand of the ith k-mer is computed. Implementation optimizations (i.e. conditional branch and buffer optimizations) can substantially reduce o_h in practice. They may depend on hardware architectures. Hence they are not discussed here.

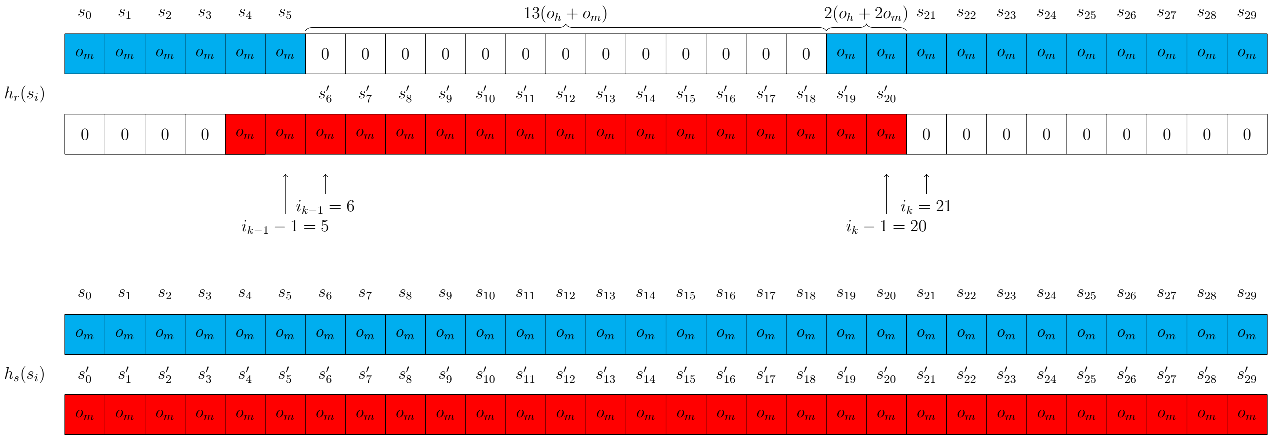

Figure 4: h_r and h_s for computing refined and standard minimizers in consecutive windows s_i (in blue) ending at base s_i and reverse complement windows s'_i (in red) ending at base s'_i, where $0 \leq i < 30$, window size $|s_i|, |s'_i| = 3$. Values in cells are operation numbers, 0 for no operation performed, o_m is for computing the order of the k-mer ending at the base.

Algorithm 1: Compute the refined minimizer in the ith consecutive window, where Q_{f*} and Q_{c*} are buffers for forward and reverse $k-$mers. $mask = 2^w - 1$

1 **Function** $h_r(s_i)$
2 **if** $\Delta_i \& 1$ **then** //Δ_i is a w-bit integer, whose jth bit is 0 iff $\delta_{i+j} > 0$. It is to compute the sign of δ_i
3 $Q_{cR}[i] \leftarrow R(\text{reverse } k\text{-mer}_i)$ //Compute and buffer the hash value of the ith reverse k-mer
4 **if** $i - w < i_{\min}$ **then** //If the last minimizer is in the current window
5 **if** $Q_{cR}[i_{\min}] > Q_{cR}[i]$ **then**
6 $i_{\min_c} \leftarrow i$ //Update the buffer if the current reverse k-mer is smaller
7 **else**
8 $i_{\min} \leftarrow i$ // Otherwise compare all the w reverse k-mers in the window
9 $j \leftarrow i - w + 1$
10 **while** $j < i$ **do**
11 **if** $Q_{cR}[i_{\min}] > Q_{cR}[j]$ **then**
12 $i_{\min_c} \leftarrow j$
13 $j \leftarrow j + 1$
14 **end**
15 **if** $\Delta_i \neq mask$ **then** //If the forward k-mer$_i$ is needed by at least one of w successor windows
16 $Q_{fR}[i] \leftarrow R(\text{forward } k\text{-mer}_i)$ //Then compute and buffer the forward k-mer$_i$
17 **if** $Q_{fR}[i] < Q_{fR}[i_{\min_f}]$ **then**
18 $i_{\min_f} \leftarrow i$ //Compare the forward k-mer$_i$ to the minimum one
19 **else**
20 $i_{min_f} \leftarrow i + 1$ //Otherwise, initiate the forward minimizer index to $i + 1$
21 $\delta_{i+w} \leftarrow \delta_{i+w-1} - p_i + p_{i+w}$ //Iterate δ_{i+w}
22 $\Delta_{i+1} \leftarrow (\delta_{i+w} \gg 63 \ll w | \Delta_i) \gg 1$ //Iterate Δ_{i+1}: Set the wth bit of Δ_{i+1} 1 if $\delta_{i+w} < 0$
23 **return** $Q_{cR}[i_{\min_c}]$, 1 //Return minimizer and the strand
24 **else** ...Q_{f*}... // Apply the same operations above to Q_{f*} except changing line 15 to $\Delta_i \neq 0$.
25 **end**

Algorithm 2: Compute the standard minimizer in the ith consecutive window, where Q_{f*} and Q_{c*} are buffers for forward and reverse $k-$mers. $sign = 2^{w-1}$

1 **Function** $h_s(s_i)$
2 $Q_{fR}[i] \leftarrow R(\text{forward } k\text{-mer}_i)$ //Compute and buffer the hash value of the ith forward k-mer
3 $Q_{cR}[i] \leftarrow R(\text{reverse } k\text{-mer}_i)$ //Compute and buffer the hash value of the ith reverse k-mer
4 **if** $Q_{fR}[i] < Q_{cR}[i]$ **then**
5 $Q_{fcR}[i] \leftarrow Q_{fR}[i]$ //Buffer the forward k-mer as the minimum k-mer if it is smaller than the reverse one
6 $\Delta_i \leftarrow \Delta_{i-1} \gg 1$ //Record the strand of the current minimum k-mer
7 **else**
8 $Q_{fcR}[i] \leftarrow Q_{cR}[i]$ //Otherwise buffer the reverse k-mer as the minimum one
9 $\Delta_i \leftarrow \Delta_{i-1} \gg 1 | sign$ //Record the strand of the current minimum k-mer
10 **if** $i - w < i_{\min}$ **then**
11 //If the last minimizer is in the current window
12 **if** $Q_{fcR}[i_{\min}] > Q_{fcR}[i]$ **then**
13 $i_{\min} \leftarrow i$ //Update the buffer if the current k-mer is smaller than the last minimizer
14 **else**
15 $i_{\min} \leftarrow i$ // Otherwise compare all the w k-mer in the window
16 $j \leftarrow i - w + 1$
17 **while** $j < i$ **do**
18 **if** $Q_{fcR}[i_{\min}] > Q_{fcR}[j]$ **then**
19 $i_{\min} \leftarrow j$
20 $j \leftarrow j + 1$
21 **end**
22 **return** $Q_{fcR}[i_{\min}]$, $\Delta_i \gg (i_{\min} - i + w - 1) \& 1$ //Return minimizer and the strand
23 **end**
